# A Dual-Network Modeling Architecture for Statistical Evaluation of College Graduates' Working Ability in Consistence with Their Job Position and Remuneration

**DOI:** 10.1155/2022/8250234

**Published:** 2022-03-07

**Authors:** Shaoyong Hong, Chun Yang, Hongwei Wen, Chao Song, Jincheng Shi, Shaohong Chen, Xiaoyu Hu

**Affiliations:** ^1^School of Data Science, Guangzhou Huashang College, Guangzhou 511300, China; ^2^School of Accounting, Guangzhou Huashang College, Guangzhou 511300, China; ^3^School of Foreign Languages, Guangzhou Huashang College, Guangzhou 511300, China; ^4^Teaching Quality Monitoring and Evaluation Center, Guangzhou Huashang College, Guangzhou 511300, China

## Abstract

Optimal human resources allocation asks to employ a person to work in the position corresponding to his/her ability. Employment competence is the key feedback to the cultivation of college students' working ability. The data relationship needs to analyze between the in-school cultivation items and the working abilities required by the companies. Machine learning framework is introduced to study the companies' responses to the cultivation of college students. In this work, a dual-network architecture is built up for statistical modeling evaluation of college graduates' working ability in consistence with their job position and remuneration. A requirement network and a cultivation network are constructed for extracting features from the original working ability data required by companies and cultivated ever in school. The networks are fully trained by adaptively tuning the linking weights. The extracted features are fused together to estimate the working competence of each target sample/person. To evaluate the dual-network model, a modeling index system is designed, including proposing a total evaluation index calculus for the dual-network model, and a variable importance index from the original data. The samples are consequently ranked by the model predicted index and by the variable importance index, respectively. The ranking difference is used to evaluate the prediction efficiency of the dual-network model. Experimental results show that the dual network architecture is feasible to establish statistical models for the evaluation of college graduates' in-school cultivated working ability in consistence with the company's required working ability at their job position and their deserved remuneration.

## 1. Introduction

The current employment competition is reflected in the contrast of the college graduates' ever-trained ability and the actual requirement of working ability by enterprises/companies. Colleges and universities should play a significant role in reforming the mode of cultivating students' basic knowledge and practical skills [1, 2]. How to guide college students to master the basic knowledge and integrate it into working skills to meet the need of company's working place should be one of the most important research subjects. The cultivation of the college students' working ability is a process influenced by many factors [3, 4]. The study can be carried out by the comparative analysis of two ends of data. The first end is the in-school training items that may conduct students to strengthen their working abilities; students have to participate in multiple professional courses and general courses, as well as the science projects, skill competitions, and part-time internships, so that they are trained to be skilled full and professionally standardized before they graduate from the colleges and universities [5, 6]. The second end is the immediate-on-board working abilities required by the companies that are willing to recruit some fresh graduates. The companies offer some job positions and corresponding remuneration, such as to specify the working field, positions, salaries to search for the high-quality talents that are probably competent for the job [7, 8]. At present, the teaching and cultivation programs of colleges and universities for college students mostly focus on the imparting of general knowledge such as science, humanities, philosophy, literature, and engineering [9], but the cultivation of the core competence of college students has not received enough emphasis on their working abilities [10]. Actually, the incorporating working ability towards different job positions is important for college students to form and develop their core competence, such as global vision, critical thinking, innovative ability, social and state responsibility, lifelong learning, entrepreneurship, and leadership coordination [11]. Collaborative filtering algorithm recommender system is one of the prevalent researched techniques to study the responses of the core competence learning to the cultivation of innovative research for college students [12]. Based on the collaborative filtering algorithm recommender system, the companies would take the “user-based” approach to estimate the college graduates' working ability, while the colleges and universities would originally take the “item-based” approach to evaluate the students' understandings. There is an evaluation gap between the “user-based” election and the “item-based” selection. Under this situation, it is necessary to pay full attention to the cultivation of the working competence of college students [13].

With the development of science and information technology, the most optimal allocation of human resources asks to employ a person to work in the position corresponding to his/her ability [14]. Then, it is much necessary to investigate intelligent algorithms to find out a shift balance between the in-school cultivated ability and the requirement of working ability from the companies. Previous studies have reported many relevant results about the college cultivation [15, 16]. The ability of learning analytics to improve the teaching processes is widely recognized [17]. Scientific innovative research has been emphasized as one of the key indicators to evaluate the graduates' abilities suiting the society requirement [18]. Machine learning frameworks have been used in data stream mining as the data information is small, weak, and discontinuous; for instance, naive Bayes and support vector machine techniques are successfully applied for document-level sentiment analysis after proper data preprocessing [19]. Dynamic big data analytical technique has been applied to investigate the ability relationships between programming and testing and thus to propose a customizable and incremental processing approach for learning analytics [20, 21]. However, the connection analysis of the college in-school cultivation and the companies' ability requirement is much inevitable for selecting talents with core working competence. There is little research concerning this point.

There is much data information in the in-school cultivation data series or in the companies' ability requirement data series. The recognition of the relationship between the in-school cultivation and the companies' requirement needs to study some advanced intelligent analytics, such as the feature extraction techniques [22], data conversion methods [23], and convolution approaches [24]. Periconceptionally, an adaptive neural network is functional to establish models to resolve the feature relationship between different data series [25, 26]. The neural network (NN) is a computational model comprised of a large number of connected nodes, each of which performs a simple calculation [27]. It performs well in dealing with the problem of non-normal distribution and is responsible for many of the recent advances in artificial intelligence [28, 29]. For example, the NN structure is widely used in identity recognition, image analysis, environmental detection, and medical diagnoses [30–33]. The criterion of optimization in the neural network is to make the error of the training set or the test set the smallest [34]. In applications, the NN model is usually designed in the way of delivering the white data feedforward and the error feedback [35]. The network linking weights are automatically trained by the input data with the data-driven machine learning mode [36].

In this paper, we employed the NN modeling mechanism to build up a dual-network modeling architecture for statistical evaluation of college graduates' working ability in consistence with their job position and remuneration. The architecture is composed of two fully connected neural networks. One is used to learn the relationship between the working ability and the requirement reflected by several companies' properties, such as working field, company scale, offered position, and affordable salary (denoted as the requirement network). The other one is used to train the connection of graduates' working ability to their ever in-school cultivation items. Important factor items are under analysis, such as the course, self-awareness, participations in the science projects, and extracurricular activities (denoted as the cultivation network). The networks will extract features from the input data by adaptively tuning the linking weights. The data features are further conducted to compute the modeling results. The output from the requirement network is used to score the ability requirement of a position, while the output from the cultivation network is used to score the students' abilities cultivated in colleges. Then, two score series were observed for all of the sample students. One represents the ability requirement from companies, and the other is to describe the college cultivation results. Consequently, these two score values were standardized and then fused together to make a dual analysis.

The neural network model is regarded as a superposition of multiple linear models [37], and then, a modeling index system is designed for evaluating the complex dual-network model. Practically, a total evaluation index is proposed to the dual-network architecture, for estimating whether students' working abilities cultivated in colleges match the companies' requirements on working competence. With fidelity to the data, an importance index is generated from the original variables involving both the in-school training items and the requirement indicators of companies. Furthermore, the target analytic samples are evaluated one by one to see if the model predicted total evaluation index matches the variable importance index. In this way, the dual-network modeling architecture is available for statistical evaluation of college graduates' working ability in consistence with the company's requirement corresponding to the relevant job positions and remunerations.

## 2. Data Acquisition

The analytical data are collected for the working people who graduated from universities and colleges in Guangdong province, China. About 3600 people were invited to participate in a survey. Their learned majors include computer, statistics, economics, arts, accounting, and they are now working in companies involving various fields such as industry, agriculture, education, IT, finance. The company scales/sizes vary from under 50 to over 3000 staffs. Some of the survey people are senior manager or junior manager, but most of them are the common working staff. Overall, their salaries range from 2 to 18 kCNY, where kCNY is an inherent currency unit representing thousand CNY.

A list of college in-school cultivation items that they have ever learned is designed in the survey questionnaire. The list includes professional courses, public courses, general courses, self-learning awareness, science projects and competitions, part-time internships and clubs. The survey people are asked to select the items which they think supporting their current working posts.

After sorting out these 3600 questionnaires, we obtained 2628 survey response data that are effective without any missing information. Then, these 2628 valid sample data are available for establishing models for statistical evaluation of college in school cultivated working ability in consistence with the people's job position and remuneration. For model establishment and optimization, the available 2628 samples were partitioned into two sample sets, respectively, for model training and testing. According to successful knowledge discovery experience, we chose 1752 samples (around 2/3) for training and 876 samples (around 1/3) for testing. The partition is performed with a random selection so as to make the training samples be objective and representative [38].

## 3. Methodologies

### 3.1. The Dual-Network Modeling Architecture

The dual-network modeling architecture is composed of two neural networks. One is for training the ability requirement based on the company's relevant properties. The other is for evaluating the students' working abilities from the college cultivation they had received. For simplicity, the former network is denoted as the requirement network (**ReqNet** for short), and the latter is denoted as the cultivation network (**CulNet** for short).

**Figure 1:**
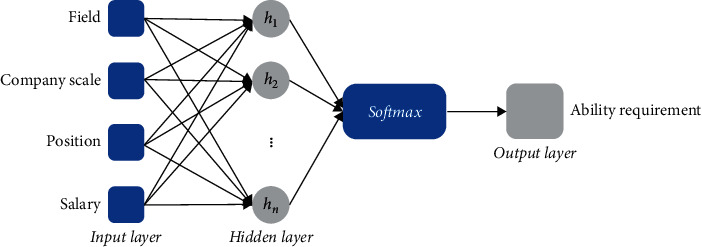
The neural network architecture for training the ability requirement based on the companies' demands on working competence.

The **ReqNet** structure is shown in Figure 1. It is a three-layer fully connected network. The neuron nodes in the input layer {*x*_*i*_*|i*=1,2,3,4} parallelly load the company's descriptive property data of its belonging field (*x*_1_), company scale (*x*_2_), offered position (*x*_3_), and affordable salary (*x*_4_). The hidden layer is designed to have *m* neural nodes (*h*_*j*_ with *j*=1,2 … *m*) for receiving the transformed data from the input layer, where *m* can be a changeable setting parameter for network training, namely,

(1) $$\begin{array}{c} h_{j}=f\left(\left(\sum_{i=1}^{4}x_{i}+b\right),j=1,2\ldots m\right), \end{array}$$

where function *f*(·) is for data activation, and *b* represents the threshold of baseline shift control. There generate *m* feature variables for establishing a network model to predict the ability requirement level.

The feature variables {*h*_*j*_} are delivered to a *Sofmax* unit, in which the norm calculus is used to score the company's requirements on working ability. Then, scorings are comprehensively stimulated by a sigmoid function, thus to output the scores (*S*_Req_) for the targeted samples, that is,

(2) $$\begin{array}{c} S_{\text{Req}}=\text{sig}\left(\sum_{j=1}^{m}{\left\|h_{j}\right\|}_{t}+c\right), \end{array}$$

where sig(·) represents the sigmoid function, ‖·‖_*t*_ stands for the *t*-norm calculus, and *c* is a threshold.

**Figure 2:**
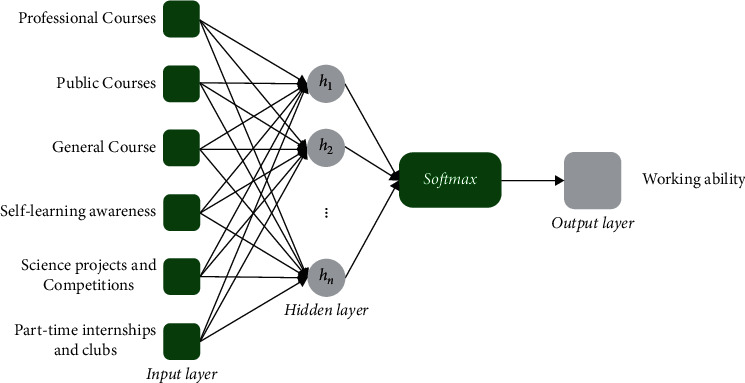
The neural network architecture for evaluating the students' working abilities trained by the college cultivation systems.

On the other hand, the **CulNet** structure is also constructed as fully connected (see Figure 2). The simulation calculations in the **CulNet** are similar to those in the **ReqNet**. The preset input nodes are available for receiving the data of in-school cultivation items when the people were undergraduates. As abovementioned, the items are statically summarized into 6 groups of professional courses, public courses, general courses, self-learning awareness, science projects and competitions, part-time internships and clubs. There corresponds to 6 input neurons (i.e., *x*_*i*_ with *i*=1,2,3,4,5,6). The hidden layer is designed to have *m* neural nodes (*h*_*j*_ with *j*=1,2 … *m*), where *m* is a tunable parameter for testing the number of hidden nodes. By network data delivery, *h*_*j*_ can be calculated as follows:

(3) $$\begin{array}{c} h_{j}=f\left(\sum_{i=1}^{4}x_{i}+b\right),\;j=1,2\ldots m. \end{array}$$

Next, the *Sofmax* unit was designed for accepting the feature data {*h*_*j*_}, and the norm calculus is also applied to score the people's in-school cultivated working ability. Then, scored data are comprehensively transformed by a logistic function to derive the output scores (*S*_Cul_) for the targeted samples, namely,

(4) $$\begin{array}{c} S_{\text{Cul}}=\text{logis}\left(\sum_{j=1}^{m}{\left\|h_{j}\right\|}_{t}+c\right), \end{array}$$

where logis(·) represents the logistic function.

To formulate the modeling index system, the total evaluation index (TEI) was proposed for estimating whether students' working abilities cultivated in colleges match the companies' requirements on working competence. It was defined as a formulating calculus related to *S*_Req_ and *S*_Cul_, that is,

(5) $$\begin{array}{c} \text{TEI}\left(i\right)=\frac{S_{\text{Req}}\left(i\right)\times S_{\text{Cul}}\left(i\right)}{e^{\left|S_{\text{Req}}\left(i\right)-S_{\text{Cul}}\left(i\right)\right|}}, \end{array}$$

where *i* goes for every targeted input sample. TEI is utilized to evaluate the job suitability of each student sample. A higher value of TEI represents there provided a more appropriate working post for a specific student. Consequently, a dual-network-triggered series is recorded in the descending order of TEI values, which is denoted as DNT series corresponding to the sample series.

### 3.2. Modeling Metrics

**Figure 3:**
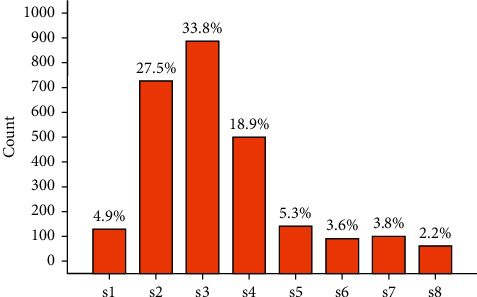
Counts of different salary levels and the standardized salary weights (*θ*) defined for each level.

Referring to the modeling index system, the dual-network modeling architecture is able to predict the TEI value of each sample for the evaluation of students' working abilities. A good prediction is expected to be suitably fitted to the major, working field, position, and salary of most targeted samples. As for model evaluation, the variable important index (VI) is proposed to make multivariate determinations. Correspondingly, the data are prearranged obeying the following rules:

(1) The data were classified in 8 working fields and 4 types of majors. We checked these working fields and majors, to evaluate the people's job suitability if their working field suits their ever studied majors. Statistically, we applied the Apriori algorithm [39] to compute the frequency of each major falling in the fields (*F*_*m*2*w*_) and, contrarily, to compute the frequency of each working field carrying the number of majorities (*F*_w2m_). In this way, the variable important index of the major fitting the working field (VI_mw_) was defined as
  (6) $$\begin{array}{c} {\text{VI}}_{\text{mw}}=F_{\text{m}2\text{w}}\times F_{\text{w}2\text{m}}. \end{array}$$
Then, the VI_mw_ was calculated for all samples and further standardized by Min-Max normalization [40]. The standardized VI_mw_ values are shown in Table 1 corresponding to each pair of major and working fields.
(2) The data were rearranged in 12 different posts with pairwise cross-defined by the 4 working types and 3 working positions (see Table 2). On this basis, the variable important index of the working post (VI_tp_) was calculated and further standardized by Max-Min normalization. The standardized VI_tp_ values are also shown in Table 2.
(3) The data were divided into 8 segments to make equivalent intervals at the salary aspect (see Table 3). Then, we counted the number of samples distributed in each of the segments. A histogram was drawn (see Figure 3) so that the column percentiles can be taken as the variable important index of each salary level (VI_s_).

Based on these three rules, a total variable important index (VI_total_) is defined for comprehensive quantitative estimation of the target people's job suitability. It can be calculated for each sample, using the convolutional cross product of VI_mw_, VI_tp_, and VI_s_, namely,

(7) $$\begin{array}{c} {\text{VI}}_{\text{total}}={\text{VI}}_{\text{mw}}⊗{\text{VI}}_{\text{tp}}⊗{\text{VI}}_{\text{s}}, \end{array}$$

where the symbol operator ⊗ represents the inner product calculation. Then, a multivariate determinant (MVD) series is defined corresponding to the descending order of VI_total_. If the DNT order matches the MVD order for all samples, we conclude that the dual network model performs 100% accurate prediction. Actually, a good prediction model can be found if the model predicted DNT series matches the MVD series on percentage. For quantitative evaluation, it is necessary to set a threshold to test the differences between DNT and MVD, as to identify the model prediction errors.

## 4. Results and Discussions

The dual-network modeling architecture is applied for the evaluation of college graduates' working ability in consistence with their job position and remuneration. 1752 training samples were used to train the **ReqNet** and the **CulNet**.

**Figure 4:**
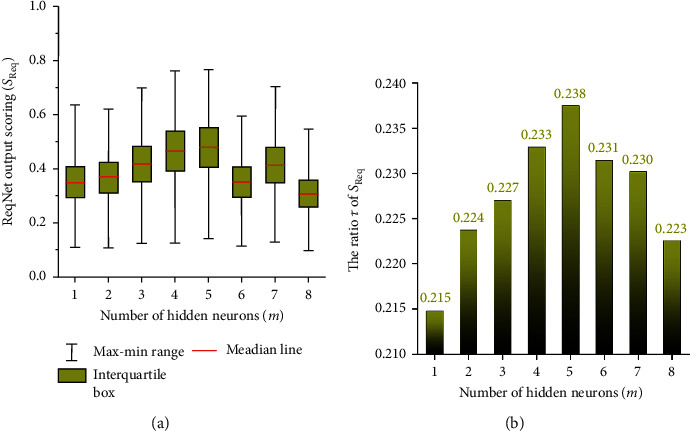
The statistical results for the output *S*_Req_ from the **ReqNet** network.

The properties of the belonging field, company scale, offered position, and affordable salary were taken as the input variables (*x*_1_, *x*_2_, *x*_3_, *x*_4_) to the **ReqNet**. The number of hidden neurons (*m*) was tuned changing from 1 to 8 (i.e., *m*=1,2 … 8), to search for an optimal **ReqNet** structure for the prediction of working ability from the requirement properties of companies. The boxplot of the outputting *S*_Req_ corresponding to the changing *m* value was shown as subfigure (a) of Figure 4. It can be learned from Figure 4(a) that the statistical data of *S*_Req_ observed the relative large max-min range at *m*=4 and *m*=5, and their quartiles cover a wider range. For detail comparison, we constructed to calculate the ratio (*τ*) of quartile range over the max-min range, which is formulated as *τ*(·)=(quartile_3_(·) − quartile_1_(·))/(max(·) − min(·)). Here, *τ*(·) is a statistical indicator to depict the main distribution range of any vector-style series. Then, *τ*(*S*_Req_) is shown in Figure 4(b). From Figure 4(b), we observed that the network with *m*=5 could have a wider main distribution range for posteriori analysis of *S*_Req_. Thus, we selected to establish the optimal **ReqNet** by using 5 hidden neurons, to extract the network features corresponding to the working ability requirement from the companies.

**Figure 5:**
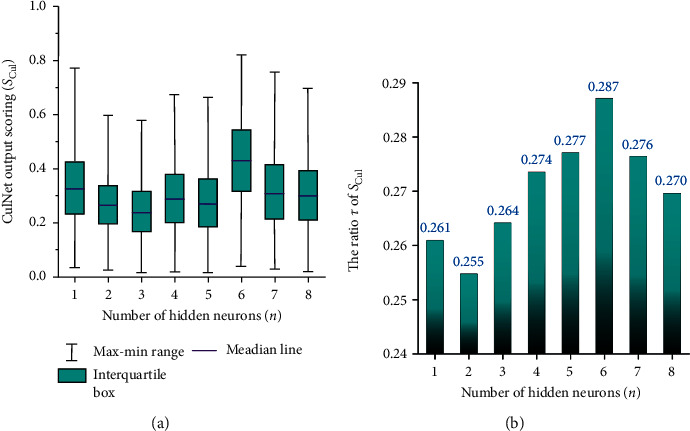
The statistical results for the output *S*_Cul_ from the **CulNet** network.

For the **CulNet** simulation, the properties of professional courses, public courses, general courses, self-learning awareness, science projects and competitions, part-time internships and clubs were taken as the input variables (*x*_1_, *x*_2_, *x*_3_, *x*_4_, *x*_5_, *x*_6_). As the coupled part of the dual network operation, the number of hidden neurons (*n*) in **CulNet** was also tuned changing from 1 to 8 (i.e., *n*=1,2 … 8), to search for an optimal structure for the prediction of the people's working ability trained from their in-school cultivation items when they were undergraduates. To predict the network output scoring series *S*_Cul_ corresponding to each value of *m*, the boxplot of *S*_Cul_ and the bar chart of *τ*(*S*_Cul_) were showed as subfigure (a) and subfigure (b) in Figure 5. The training results in Figure 5 indicated that the training of **CulNet** is able to get a wider main distribution range for posteriori analysis of *S*_Cul_ when *m*=6. Thus, we established the optimal **CulNet** by using 6 hidden neurons, to extract the network features representing the people's in-school cultivated ability when they were undergraduates.

In summary, the optimal dual network architecture was conclusively built up by a fully connected **ReqNet** composed of 5 hidden neurons coupled with a fully connected **CulNet** composed of 6 hidden nodes. The tuning of the network linking weight was designed to obey the auto self-adaptive network training mechanism. Then, the optimal architecture was applied to predict the *S*_Req_ series and the *S*_Cul_ series for the 876 testing samples.

**Figure 6:**
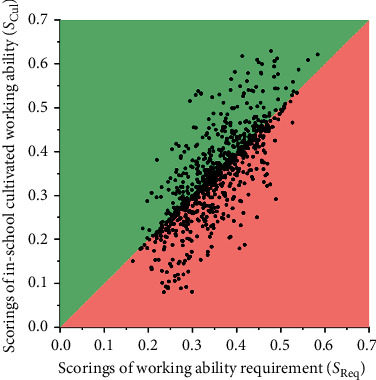
The scatter plot of *S*_Req_ versus *S*_Cul_ for the 876 testing samples.

The scatter plot of *S*_Req_ versus *S*_Cul_ is shown in Figure 6. We can easily find from Figure 6 the samples located near the 45° line. These samples have their *S*_Cul_ values close to their *S*_Req_ values, which indicated that these persons have the corresponding in-school trained ability matching their working ability required by the companies. The samples located far from the 45° line indicated that the persons are over competent (in the green area) or less qualified (in the red area) for their working positions. In this way, the dual-network modeling architecture is functional to evaluate the matching levels of the target people that are working at a right job position.

**Figure 7:**
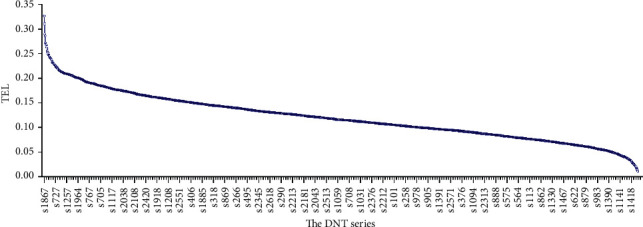
The DNT series for all of the 2628 samples based on the descending TEI values.

Furthermore, the optimal dual-network architecture was used to predict the scores of *S*_Req_ and *S*_Cul_ for all of the 2628 samples, and then, the TEI index was conducted for each sample (see Figure 7). Based on the TEI value, the samples were sorted as the DNT sample series. It is obtained from Figure 7 that the persons stand in the front of the DNT queue are much competent for their works while the persons stand at the end are less qualified for their current working positions.

**Figure 8:**
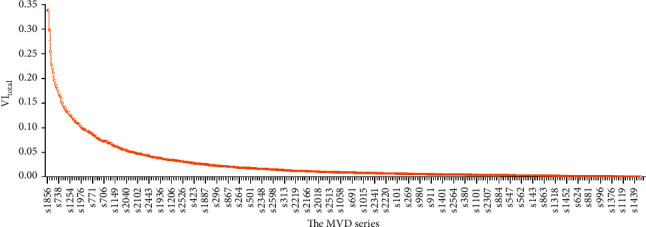
The MVD series for the 2628 samples based on the descending VI_total_ values.

On the other hand, we calculated the VI values for each of the 2628 samples. The VI_total_ value was taken as the multivariate metric for evaluating the optimized dual-network architecture because the VI calculus is designed originating from the inherent data properties of working fields, majors, working types, positions, and remuneration salaries. By the descending order of VI_total_, we observed the MVD series for all samples (see Figure 8). The MVD-order series delivered the objective evaluation values for the people who are much competent or less qualified for their current works. The persons stand in the front are regarded as competent while the persons stand at the end are taken as less qualified.

**Figure 9:**
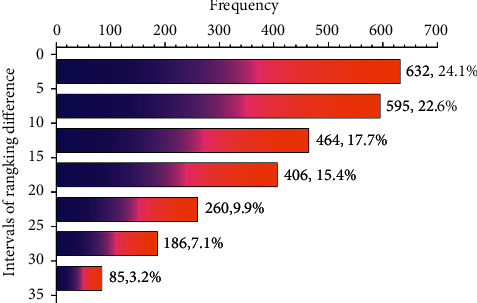
Histogram statistical results for the ranking difference between the DNT series and the MVD series for each sample.

The ranking of samples in the DNT-order series predicted by the dual network architecture was not totally the same as in the reference MVD-order series. Thus, the DNT series was compared to the MVD series for each of the 2628 samples, and the ranking difference was identified (see Figure 9). From Figure 9, we have learned that the largest difference just goes to 35 rankings, taking only 1.33% of the series rank of 2628. The result indicated that the dual network prediction of college graduates' working ability was much coincident with their job positions and remunerations.

## 5. Conclusions

In this paper, a dual-network modeling architecture was built up for statistical evaluation of college graduates' working ability in consistence with their job position and remuneration. The architecture is composed of **ReqNet** and **CulNet**. The extracted features were scored in a single *Softmax* unit with the sigmoid function (for **ReqNet**) and the logistic function (for **CulNet**). As to find the most matching of students' in-school cultivated ability to the company's working ability requirement, the scorings of *S*_Req_ and *S*_Cul_ were further fused to calculate the TEI index value. The descending order of TEI was used to evaluate the dual-network model prediction to sort the matching degree of the students' working ability. Then, we observed the DNT sample series (see Figure 7) and identified the over competent and the less qualified persons (see Figure 6). To evaluate the model prediction capacity, the VI values were calculated concerning on some key properties of the original data. In detail partitions, VI_mw_ was determined by the properties of working fields and majors, VI_tp_ by the working types and positions, and VI_s_ by the salary and remuneration. Then, the inner product indicator VI_total_ was taken as a fusion factor to comprehensively evaluating the variable importance. Then, the MVD sample series was observed in the descending order of VI_total_ (see Figure 8). Concerning on the ranking difference of DNT and MVD, the evaluation of in-school cultivated working ability in consistence with their position-required ability was much accurate, with the largest ranking difference taking only 1.33% of the whole ranking length. Furthermore, the distribution of ranking difference (see Figure 9) showed that there are most samples (64.4%) going with small different rankings (less than 15), appropriate number of samples (25.3%) with moderate difference (over 15 but less than 25), and seldom samples (10.3%) with over 25 different rankings. These modeling and comparing results indicated that the dual network model is reasonable to get accurate prediction results for statistical evaluation of college graduates' in-school cultivated working ability in consistence with the company's required working ability at current job position and their deserved remuneration.

## Figures and Tables

**Table 1:** The standardized VI_mw_ of job suitability based on the major type suiting the working fields.

Working fields	Major type			
	Technology	Language	Marketing	Management
Heavy industry	0.6516	0.1000	0.2452	0.2452
Light industry	0.5355	0.1290	0.3032	0.1581
Agriculture, food, & chemistry	0.7677	0.1581	0.4774	0.4194
Economics & finance	0.2452	0.2161	0.7677	1.0000
Education & medical	0.8839	0.1871	0.1871	0.3323
Services & administration	0.1290	0.6806	0.1290	0.8839
Delivery & IT communication	0.7677	0.2452	0.4194	0.6516
Religious belief & entertainment	0.1000	1.0000	0.3613	0.5935

**Table 2:** The standardized VI_tp_ for the working position of different working types.

Working type	Working position	The standardized VI_tp_
Management	Staff	0.3455
Management	Junior manager	0.6727
Management	Senior manager	1.0000
Technology	Staff	0.2636
Technology	Junior manager	0.5091
Technology	Senior manager	0.7545
Administration	Staff	0.1000
Administration	Junior manager	0.1818
Administration	Senior manager	0.2636
Marketing	Staff	0.1818
Marketing	Junior manager	0.3455
Marketing	Senior manager	0.5091

**Table 3:** The standardized VI_s_ of the salary paid.

	Salary paid range (unit: kCNY)							
	[2,4)	[4,6)	[6,8)	[8,10)	[10,12)	[12,14)	[14,16)	[16, 18]
Salary level	s1	s2	s3	s4	s5	s6	s7	s8
Amount/count	129	723	888	497	139	94	99	59
The standardized VI_s_	0.049	0.275	0.338	0.189	0.053	0.036	0.038	0.022

## Data Availability

The data used to support the findings of this study are available from the corresponding author upon request.
